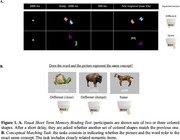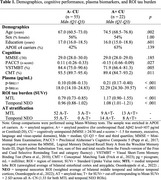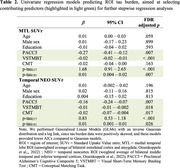# Targeting pre‐symptomatic AD individuals: Deep cognitive assessment and plasma biomarkers to predict tau aggregation

**DOI:** 10.1002/alz70856_098968

**Published:** 2025-12-24

**Authors:** Lisa Quenon, Lara Huyghe, Jean‐Louis Bayart, Emilien Boyer, Lise Colmant, Yasmine Salman, Thomas Gérard, Vincent Malotaux, Emma Delhaye, Gabriel Besson, Laurence Dricot, Renaud Lhommel, Adrian Ivanoiu, Christine Bastin, Bernard J Hanseeuw

**Affiliations:** ^1^ Saint‐Luc University Hospital, Brussels, Brussels, Belgium; ^2^ Institute of Neuroscience, UCLouvain, Brussels, Belgium; ^3^ Institute of Neuroscience, UCLouvain, Brussels, 1200, Belgium; ^4^ Departement of Laboratory Medicine Cliniques Saint Pierre, Ottignies, Belgium; ^5^ Grand Hôpital de Charleroi, Charleroi, Belgium; ^6^ Saint‐Luc University Hospital, Brussels, Belgium; ^7^ Massachusetts General Hospital, Harvard Medical School, Boston, MA, USA; ^8^ GIGA‐CRC, University of Liège, Liège, Belgium

## Abstract

**Background:**

Cognitively unimpaired (CU) individuals with both elevated brain amyloid load and tau burden in the medial temporal (MTL) and temporal neocortex (NEO) face high risk of short‐term cognitive decline (≤5 years; risk=50%). However, identifying these individuals in the population remains challenging due to their low prevalence (8‐10%), the cost and invasiveness of validated biomarkers. Cognitive measures and blood‐based biomarkers offer promising scalable alternatives, but most plasma biomarkers are more closely associated with amyloid than tau aggregates, and tau‐specific measures remain poorly defined. This study assessed whether specific cognitive tasks, including tasks targeting the functions of the first affected regions by tauopathy, and blood‐based biomarkers can predict early tau aggregation.

**Method:**

Seventy‐seven CU participants completed the Visual Short‐Term Binding Test (VSTMBT), the Conceptual Matching Task (CMT), the cognitive tests required for the Preclinical Alzheimer's Cognitive Composite (PACC5), a blood‐test, [^18^F]‐MK6240 tau‐PET imaging, 3T‐MRI, and amyloid (A) status determination (A+ for Centiloid≥ 20 or cerebrospinal fluid amyloid‐beta_42_≤437 pg/mL). The VSTMBT and CMT (Figure 1) involve fined‐grained perceptual and conceptual discrimination, respectively, supposedly relying on the transentorhinal cortex. The sample included 55 A‐ CU and 22 A+ CU (Table 1). Standard Uptake Value ratios (SUVr) were computed for MTL and temporal NEO region of interests (ROI; Ossenkoppele et al., 2022; reference=grey cerebellar). Plasma *p*‐tau_217_ and *p*‐tau_181_ levels were quantified using Lumipulse and SIMOA. Univariate regression models predicting ROI tau burden based on demographics (age, sex, education), cognitive performance (VSTMBT, CMT, PACC5), and plasma *p*‐tau species (2 ROIs x 8 predictors) were conducted to select contributing predictors (highlighted in green in Table 2) for further stepwise regression analyses (both directions).

**Result:**

For MTL tau burden, optimal model fit (initial AIC=4.88, final AIC=3.55) was found with the VSTMBT (*b* = ‐0.01, SE=0.004, *p* = .004) and plasma *p*‐tau_217_ level (*b* = 1.18, SE=0.276, *p* <.001) as predictors. For temporal NEO tau burden (initial AIC=41.04, AIC=‐44.92), best fit was found with the PACC5 (*b* = ‐0.09, SE=0.04, *p* = .027) plasma *p*‐tau_217_ (*b* = 0.77, SE=0.15, *p* <.001; *b* = 0.76) and *p*‐tau_181_ (*b* = ‐0.003, SE=0.002, *p* = .125) levels as predictors.

**Conclusion:**

Plasma *p*‐tau_217_ predicted tau burden across both ROIs, alongside different cognitive tasks depending on the ROI, likely reflecting their associated cognitive functions.